# Oxalate-mediated detoxification and bioaccumulation of lead and cadmium in Escherichia coli K-12 MG1655

**DOI:** 10.1099/mic.0.001728

**Published:** 2026-07-06

**Authors:** Nnabueze Darlington Nnaji, Chukwudi U. Anyanwu, Taghi Miri, Helen Onyeaka

**Affiliations:** 1 Department of Microbiology, University of Nigeria, Nsukka 410105, Nigeria; 2 School of Chemical Engineering, University of Birmingham, Birmingham B15 2TT, UK

**Keywords:** bioaccumulation, biorecovery, bioremediation, cadmium, *Escherichia coli*, lead, oxalic acid

## Abstract

Lead (Pb) and cadmium (Cd) remain among the most persistent and hazardous heavy-metal contaminants in industrial effluents, posing severe risks to ecosystems and human health due to their non-biodegradable nature and high toxicity. In response to the limitations of conventional chemical remediation technologies, this study evaluates the potential of *Escherichia coli* K-12 MG1655 to function as a microbially driven system for the detoxification, sequestration and recovery of Pb and Cd. Emphasis is placed on oxalic acid production as a mechanistic basis for metal tolerance. HPLC confirmed that *E. coli* K-12 MG1655 synthesizes oxalic acid under metal stress, with Pb exposure eliciting the better oxalate output, providing evidence consistent with metal–oxalate-associated detoxification during metal stress. Bioaccumulation studies using inductively coupled plasma–optical emission spectrometry revealed exceptional metal removal efficiencies, reaching 99.94% for Pb and 97.77% for Cd at 1,000 p.p.m., while Pb+Cd mixed-metal systems maintained high overall uptake (98.19%). These results demonstrate that *E. coli* can sequester metals across a wide concentration range with minimal inhibition from competitive ion interactions. Metal recovery from loaded biomass was evaluated through acid desorption and ohmic heating. Nitric acid (0.1 M HNO_3_) achieved the highest recovery efficiencies (Pb, 98.5%; Cd, 91.5%), whereas ohmic heating yielded moderate (Pb, 45.38%; Cd, 45.83%) but environmentally favourable recovery without chemical additives. The integrated findings illustrate a complete microbial remediation–recovery cycle encompassing detoxification via oxalic acid, high-efficiency metal sequestration and effective downstream recovery. This integrative study establishes *E. coli* K-12 MG1655 as a promising candidate for closed-loop bioremediation systems linking detoxification, sequestration and recovery of heavy metals.

**Impact Statement**

This study addresses a major gap in microbial bioremediation research by integrating the interconnected processes of detoxification, metal bioaccumulation and metal recovery within a single microbial platform. By demonstrating that oxalic-acid-driven detoxification directly enhances bioaccumulation performance and enables subsequent metal release through either dilute acid or ohmic heating regeneration, this work provides a unified framework linking microbial physiology with practical recovery technologies. The study advances the field by showing how microbial systems can be engineered into circular, regenerable bioprocesses, reducing dependency on chemically intensive methods and offering scalable, sustainable solutions for the remediation of metal-contaminated environments.

## Introduction

Heavy metal contamination continues to pose a serious global threat to the environment and public health, with the persistence and toxicity of these metals contributing to increasing ecological crises across both industrialized and developing regions. According to the World Health Organization [1], heavy metal pollution is one of the key public health issues, particularly in low-income countries where poor management of industrial waste often subjects the population to polluted soil and water. Regulatory frameworks, such as the European Union Water Framework Directive [2] and United States Environmental Protection Agency priority pollutant listings [3], underscore the international recognition of Pb and Cd as high-risk toxicants. Pb is considered the most dangerous contaminant because of its neurotoxicity, and Cd has severe renal, skeletal and carcinogenic effects and is ranked sixth on the Agency for Toxic Substances and Disease Registry toxicity list [4]. These issues are not far-fetched, empirical measurements in highly industrialized regions like the Niger Delta have found that the concentration of Pb and Cd in residents reaches up to six times the acceptable limit, further increasing the cumulative human-health burden of chronic exposure [5]. These cases highlight the scale of the contamination problem and support the importance of new approaches that should be sustainable and socio-economically available.

The traditional heavy-metal removal methods, such as chemical precipitation, ion exchange and electrochemical methods, have been widely used but are limited by the high cost of operation, partial recovery of metals and generation of toxic sludge [6]. These constraints depict the inefficient nature of the remediation measures that are based only on physicochemical principles, especially when used in complex effluent matrices or in low concentrations of metal ions. The further increase in industrialization, urbanization and mining has also increased anthropogenic metal emissions [7], which exceeds the ability of traditional technologies to provide effective environmental protection. As a result, the focus of research has changed to more sustainable, biologically inspired methods that would be able to tackle ecological and economic limitations. The new paradigm focuses on the resource circularity, low-energy needs and ecologically friendly metal detoxification and recovery pathways.

Bioremediation has, therefore, become a trend, which is an environmentally friendly alternative that is based on living organisms, particularly micro-organisms that are able to accumulate, transform or immobilize the toxic metals [8]. *Escherichia coli* has been distinguished in this field due to its wide metabolic flexibility, genetic plasticity and quick adaptation to environmental stresses. Past research shows that the interaction of *E. coli* with a wide range of heavy metals can modulate biosorption through cell wall components, transporter systems and stress-related biochemical pathways [9]. Another factor that demonstrates bacterial metabolic flexibility is the release of organic acids in response to toxic stress, such as oxalic acid, a metabolite that can bind metal ions to form stable, insoluble oxalate complexes [10]. This is commonly known as microbial detoxification, which reduces reactive oxygen species, ensures metal homeostasis and reduces cellular toxicity [11]. Similar results are reported in parallel studies involving microbial strains that are exposed to Pb, which increases the production of oxalic acid [12]. This supports the general hypothesis that organic acid production is an evolutionary response to oxidative and ionic stress.

Although significant progress has been made, the existing literature has a disjointed perception of how the processes of detoxification, metal uptake and biorecovery overlap in a single microbial system. Most existing studies on heavy metal removal focus on either the efficiency of biosorption [13,14], the metal-tolerance mechanisms [15] or desorption strategies [16] in isolation, with limited attention to how these steps can be integrated into a single, regenerable process. This siloed approach obscures how metabolic responses influence metal sequestration efficiency or how intracellular and extracellular detoxification pathways may determine the feasibility of downstream metal recovery. Although the uptake of heavy metals has been described in systems, little has been done to examine the dynamic nature of the metabolites, such as oxalic acid, in determining the sequestration and recovery potential. In addition, although acid desorption has proven to be an efficient recovery technique [17], other techniques, including ohmic heating, which is a relatively new technology, are under-researched and not well incorporated into the circular bioremediation framework.

The lack of integrative studies leaves several critical questions unanswered. How does *E. coli* coordinate cellular defences, including membrane stabilization, extracellular polymeric substance (EPS) restructuring, enzymatic protection and organic acid secretion in response to metal stress? What is the relationship between oxalic acid production and metal bioaccumulation efficiency under single and mixed metal exposure? Most importantly, what can be done to use these biological processes to enable successful metal biorecovery and, in turn, lessen the use of chemically intensive approaches and enable sustainable circular economies? Addressing these gaps is essential to further the science of microbial bioremediation and to attain scalable, real-world solutions that can ameliorate the heavy metals contamination globally.

Oxalate-associated detoxification in *E. coli* K-12 appears to operate as part of a broader stress-responsive metabolic adjustment rather than a single dedicated ‘oxalate pathway’. In *E. coli* K-12, exposure to pollutants including heavy metals is known to activate global regulators, including *SoxRS* and *OxyR*, which coordinate defences against oxidative stress, metal redox cycling and intracellular ion imbalance [18,19]. These responses converge with carbon flux redistribution mediated by *PoxB* and central metabolic regulators, promoting secretion of low-molecular-weight organic acids [20]. Such organic acids have repeatedly been shown to chelate and immobilize divalent cations, reducing intracellular toxicity and stabilizing cellular redox status [21]. Although *E. coli* does not possess a canonical fungal-like oxalate biosynthesis pathway, oxalate formation in bacteria has been associated with oxidation of glyoxylate and stress-linked overflow metabolism [22,23], supporting the biological plausibility of oxalate secretion during metal challenge.

Importantly, several transcriptomic studies [24,26] demonstrate predictable up-regulation of acid-stress, oxidative-stress and small-molecule export systems during heavy metal exposure, indicating the likelihood that oxalate secretion is regulated and reproducible rather than incidental. These regulatory drivers support the rationale for selecting *E. coli* K-12 MG1655, a genetically stable and mechanistically transparent strain, as a model platform for biotechnology applications where predictable physiological behaviour under stress is desirable.

Here, we establish an integrated platform based on non-pathogenic *E. coli* K-12 that (i) employs oxalic-acid-mediated detoxification to maintain viability at Pb and Cd concentrations up to 1000 mg l^−1^, (ii) delivers high-efficiency bioremoval of Pb and Cd in single and binary metal systems and (iii) evaluates both dilute nitric acid and ohmic heating as regeneration routes for metal recovery. To our knowledge, few studies have examined oxalate-associated detoxification, metal sequestration and downstream regeneration within a single bacterial remediation platform. Although microbial organic-acid production, biosorption and metal desorption have individually been reported in the literature, these processes are commonly investigated in isolation. Consequently, the mechanistic relationship between stress-responsive metabolite production, metal sequestration performance and biomass regeneration remains insufficiently integrated. The present study, therefore, focuses primarily on establishing a unified remediation–recovery framework in which detoxification-associated oxalate production is examined alongside bioaccumulation and post-treatment metal recovery.

Therefore, this study aims to elucidate the detoxification, bioaccumulation as well as biorecovery of Pb- and Cd-exposed *E. coli* K-12 MG1655, including oxalic-acid-mediated tolerance and comparative study of acid and ohmic heating recovery processes. Through a unified examination of microbial physiology, metabolic responses and recovery pathways, this work seeks to advance a more holistic understanding of microbial metal processing and contribute to the development of sustainable, circular bioremediation frameworks.

## Methods

### Bacterial culture and heavy metal exposure

A laboratory strain of *E. coli* K-12 MG1655 was obtained from the microbial culture collection of the Biochemical Engineering Laboratory, School of Chemical Engineering, University of Birmingham. The strain was revived by transferring a lyophilized bead into nutrient broth and incubated at 37 °C and 150 r.p.m. within 24 h. Purity of culture was established through streak plating on nutrient agar. To achieve physiological homogeneity in the presence of metal, the culture was allowed to reach mid-logarithmic growth and was spectrophotometrically monitored at 600 nm with OD_600_ values between 0.5 and 1.0 considered suitable for the experiment. Cultures with a value above this were diluted accordingly.

Analytical-grade lead nitrate [Pb(NO_3_)_2_] and cadmium nitrate tetrahydrate [Cd(NO_3_)_2_·4H_2_O] were used as the sources of metals. Stock solutions were made by dissolving 1.6 g Pb(NO_3_)_2_ and 2.74 g Cd(NO_3_)_2_·4H_2_O in 1 l of deionized water; 100 p.p.m., 200 p.p.m. and 500 p.p.m. stock solutions were prepared by diluting the stock solutions. A 0.1 M NaOH or HCl was added to buffer the metal solutions to pH 7.0 and counter the pH-dependent changes in metal bioavailability.

Mineral salt medium (MSM) was composed of 1.73 g l^−1^ K_2_HPO_4_, 0.1 g l^−1^ MgSO_4_·7H_2_O, 1 g l^−1^ NH_4_NO_3_, 0.03 g l^−1^ FeSO_4_·7H_2_O and 5 g l^−1^ yeast extract. For each assay, 1 ml inoculum was inoculated in a solution of 40 ml MSM, 20 ml sterile glucose solution and 40 ml metal solution. The metal-free control consisted of 80 ml MSM and 20 ml glucose solution. Cultures were incubated at 37 °C and agitated in a rotary shaker at 150 r.p.m. for 24 h to achieve uniform aeration and metabolic activity in the presence of metals.

Culture pH was monitored throughout incubation using a calibrated digital pH metre to evaluate acidification associated with microbial metabolism and metal exposure. Additional tolerance assays were performed to determine the maximum Pb and Cd concentrations permitting detectable bacterial viability over 24-h incubation. Viability was assessed by Log_10_ c.f.u. measurements across increasing metal concentrations.

### Quantification of oxalic acid

To ensure that differences in metabolite profiles were not due to unequal biomass accumulation, cultures were grown to comparable mid-log phase and normalized to equivalent OD_600_ values prior to metal exposure. HPLC was conducted on the 1,000 p.p.m. treatment using an Agilent 1260 Infinity II system (Santa Clara, CA, USA) to explain the production of oxalic acid as a possible detoxification product. After 24 h, culture samples treated with Pb and Cd were centrifuged to remove biomass; the supernatants were filtered with 0.22 µm membranes to remove any residual particulates that could affect chromatographic resolution. The standard of oxalic acid at 50 p.p.m. was prepared and analysed to determine the reference retention time and the corresponding peak area, which defines the calibration baseline required to perform absolute quantification. Reverse-phase C18 columns (250 mm × 4.6 mm, 5 um) were used with flow rates of 1.0–1.2 ml min^−1^ and UV detection at 240, 280, 320 and 350 nm. The mobile phase mixture was 85% acetonitrile and 15% water. The identity of the peaks in chromatograms of experimental samples was verified against the standard; the retention times were used as the main diagnostic criterion. The calibration curve was obtained using the 50 p.p.m. standard, and the areas of the peaks were determined and quantified to obtain the concentration of oxalic acid in each sample.

### Fourier-transform infrared characterization of secreted oxalic acid following 1,000 p.p.m. Pb exposure

Fourier-transform infrared characterization (FTIR) was used to characterize oxalic acid synthesized by *E. coli* K-12 MG1655 exposed to 1,000 p.p.m. Pb. After 24-h exposure to metals, centrifugation was done using a Sigma Refrigerated 2023 (1320 /G13) centrifuge at 10,000×g for 20 min at room temperature to separate the bacterial cells and take the supernatant, which contained secreted oxalic acid. Fourier transform infrared spectra were recorded with a Bruker Vertex 80 FTIR spectrometer with an attenuated total reflectance accessory. Acquisition of the spectra was done at a wavenumber range of 4,000–650 cm^−1^.

### Bioaccumulation studies

Bioaccumulation of Pb and Cd by *E. coli* was quantified using the Perkin Elmer Optima 8000 inductively coupled plasma–optical emission spectrometry (ICP-OES). Cultures were centrifuged after 24-h exposure to metals to separate supernatant and bacterial pellets. Supernatants were retained for residual metal analysis, and pellets were digested in acid to recover metals associated with the biomass fraction, including intracellularly accumulated, surface-bound and potentially co-precipitated metal species. Concurrent ICP-OES of the two fractions gave accurate measurements of metal uptake. Bioaccumulation efficiency was calculated using the established equation:


$$\text{Bioaccumulation Efficiency (\%)}=\frac{C_{i}-C_{f}}{C_{i}}\times 100$$

where $C_{i}$ represents the initial metal concentration and $C_{f}$ is the final concentration in the supernatant. Experiments were conducted in both single-metal and mixed-metal systems to investigate competitive interactions.

### Biorecovery studies

Biorecovery experiments were used to test the viability of recovering Pb and Cd in metal-contaminated biomass of *E. coli* K12 MG1655 using two different techniques: acid-based desorption and ohmic heating. This experiment was carried out for the 200 p.p.m. setups. A 15 ml culture containing *E. coli* biomass from the test setup after 24 h was centrifuged at 10,000 ***g*** for 10 min. The pellet was collected and thoroughly washed with PBS to remove any loosely bound heavy metals on the surface. Acid solutions of HCl at 0.01 M, 0.1 M and HNO_3_ at 0.01 M and 0.1 M were prepared in sufficient quantities. After decanting the supernatant and leaving the *E. coli* biomass, 15 ml of either 0.01 M or 0.1 M HCl, or 0.01 M or 0.1 M HNO_3_, was added. The biomass was fully immersed in the acid solution and placed in a shaker to gently mix the solution, promoting effective contact between the biomass and the acid. The biomass was incubated in the acid solution for 1 h at room temperature. After the incubation period, the biomass was separated from the acid solution by filtration. The supernatant, now containing the desorbed heavy metals, was collected in a clean container for ICP-OES analysis.

The second method of biorecovery involved ohmic heating. A resuspension of 50 ml of deionized water of the biomass of *E. coli* was made in a three-necked, 100-ml glass reactor with two stainless-steel electrodes 4.25 cm apart. The ohmic heating system was operated following the procedure described by Chu *et al*. [27]. The setup was operated at 30 V, with the temperature maintained at about 30–35 °C over a 10-min period. This created cell disruption and release of metals without the use of chemical reagents, hence minimizing secondary waste. Further filtration provided a supernatant that was subjected to ICP-OES. Recovery efficiency for each method was calculated using the formula:


$$\text{Recovery Efficiency (\%)}=\frac{C_{r}}{C_{t}}\times 100$$

where $C_{r}$ is the concentration of recovered metal and $C_{t}$ is the total metal content initially present in the biomass.

### Statistical analysis

Experiments were conducted in triplicate, and the data obtained were subjected to statistical analysis to ensure reliability and reproducibility. ANOVA was used to identify significant differences in treatment means with special focus on comparing the bioaccumulation rates and recovery efficiencies under experimental conditions. Where ANOVA identified significant variation, least significant difference (LSD) tests were used to identify pairwise differences at the confidence level of *P*<0.05.

## Results

### Maximum tolerance concentration of *E. coli* under Cd and Pb stress

*E. coli* K-12 MG1655 exhibited substantial tolerance to both Cd and Pb exposure across a broad concentration range (Tables1  2). Under Cd stress, viable growth was maintained up to 2,000 p.p.m., although progressive reductions in viability were observed at concentrations ≥1,200 p.p.m. No viable growth beyond the initial time point was detected above 2,000 p.p.m. Cd. Under Pb exposure, *E. coli* remained viable up to 2,400 p.p.m., with viability progressively declining at concentrations ≥1,600 p.p.m. and complete loss of detectable growth after prolonged incubation at ≥1,200 p.p.m.

**Table 1.** Viability of *E. coli* K-12 MG1655 under increasing Cd concentrations

Time (hr)	Control	400 p.p.m.	600 p.p.m.	800 p.p.m.	1,200 p.p.m.	1,600 p.p.m.	2,000 p.p.m.
0	8.12±0.51^a^	7.97±0.18^a^	7.94±0.43^a^	7.96±0.89^a^	6.58±0.55^b^	6.90±0.34^b^	6.61±0.20^b^
2	8.17±0.67^a^	8.10±0.56^a^	8.02±0.37^a^	8.07±0.90^a^	6.88±0.09^b^	5.69±0.83^b^	0
5	10.03±0.43^a^	9.10±0.54^b^	9.02±0.28^b^	9.07±0.69^b^	6.86±0.44^c^	0	0
24	10.04±0.71^a^	9.10±0.56^b^	9.00±0.60^b^	9.08±0.11^b^	5.98±0.23^c^	0	0

Results are reported as mean±sd; mean values are in Log_10_ c.f.u., *N*=3. Different superscript letters within each treatment indicate significant differences (LSD, *P*<0.05). *E. coli* growth declined with increasing Cd (400-2,000 ppm) and exposure time; no viable cells remained at 2,000 p.p.m. after 2 h or at 1,600-2,000 p.p.m. after 5 h.

**Table 2.** Viability of *E. coli* K-12 MG1655 under increasing Pb concentrations

Time (hr)	Control	600 p.p.m.	800 p.p.m.	1,200 p.p.m.	1,600 p.p.m.	2,000 p.p.m.	2,400 p.p.m.
0	8.12±0.51^a^	7.76±0.67^a^	8.00±0.33^a^	8.01±0.59^a^	7.02±0.63^b^	6.64±0.18^c^	6.63±0.09^c^
2	8.17±0.67^a^	8.05±0.43^a^	8.06±0.37^a^	8.04±0.56^a^	6.91±0.44^b^	5.60±0.82^c^	0
5	10.03±0.43^a^	9.09±0.52^b^	9.08±0.82^b^	7.92±0.48^c^	6.18±0.56^d^	5.00±0.61^e^	0
24	10.04±0.71^a^	9.13±0.21^b^	9.11±0.23^b^	0	0	0	0

Results are reported as mean±sd; mean values are in Log_10_ c.f.u., *N*=3. Different superscript letters within each treatment indicate significant differences (LSD, *P*<0.05). *E. coli* growth declined with increasing Pb concentration and exposure time. No viable cells remained at 2,400 ppm after 2 h or ≥1,200 ppm after 24 h.

### Oxalic acid production under metal stress

**Fig. 1.**
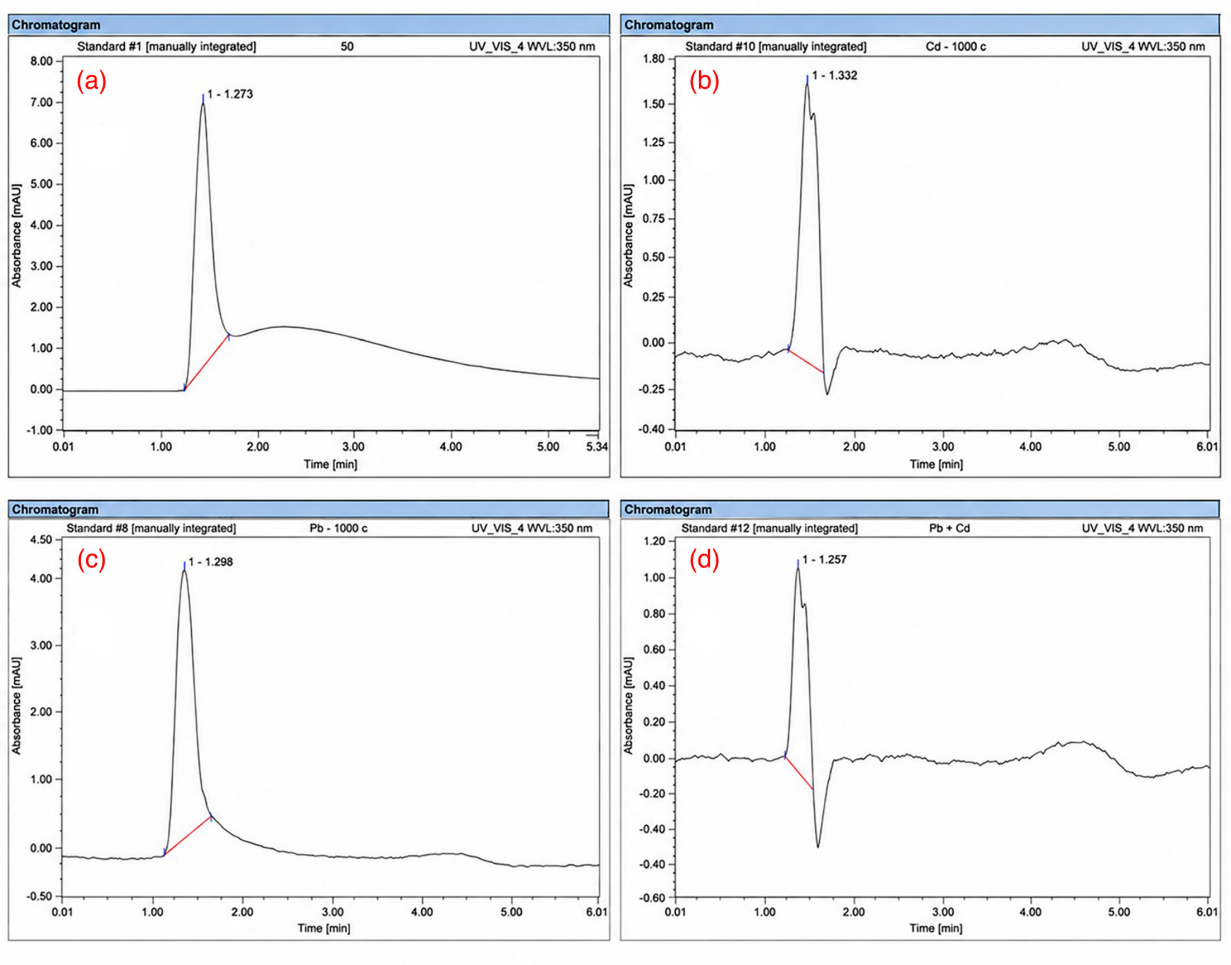
HPLC chromatogram of (a) known oxalic acid standard (50 p.p.m.); (b) oxalic acid from *E. coli* exposed to 1,000 p.p.m. Pb; (c) oxalic acid from *E. coli* exposed to 1,000 p.p.m. Cd; (d) oxalic acid from *E. coli* exposed to 1,000 p.p.m. Pb+Cd. Four HPLC chromatograms showing oxalic acid peaks with comparable retention times and varying peak intensities.

Quantification of oxalic acid in cultures of *E. coli* K-12 MG1655 exposed to Pb (1,000 p.p.m.) and Cd (1,000 p.p.m.) and their combination was determined using HPLC. A 50 p.p.m. standard of oxalic acid was used to compare samples to detect oxalic acid produced by *E. coli* under metal stress based on retention times, peak areas and peak heights. The known standard had a retention time of 1.273 min, with a peak area of 1.135 mAU min, and a peak height of 6.313 mAU (Fig. 1a). This result served as a reference point for the subsequent analysis of *E. coli* samples.

The retention time in cultures exposed to 1,000 p.p.m. Pb was slightly delayed at 1.298 min, the peak area was 0.937 mAU min and the peak height was 3.831 mAU (Fig. 1b). On the other hand, the retention time of 1,000 p.p.m. Cd was 1.332 min with the peak area being significantly smaller (0.345 mAU min) and the peak height (1.696 mAU) being lower (Fig. 1c). The culture exposed to the combined stress of Pb and Cd (1,000 p.p.m. each) had the shortest retention time of 1.257 min, the lowest peak area of 0.217 mAU min and a peak height of 1.086 mAU (Fig. 1d). These findings indicate the presence of measurable oxalic acid associated with metal stress exposure, and the levels of production differ depending on the type of metal exposure. The highest oxalic acid production was observed in the 1,000 p.p.m. Pb condition as indicated by the larger peak area and height compared to 1,000 p.p.m. Cd or the 1,000 p.p.m. Pb+Cd exposure, suggesting that Pb exposure may induce a stronger oxalate-associated metabolic response, which includes oxalic acid production *in E. coli*.

### pH changes during Cd and Pb exposure

Progressive decreases in culture pH were observed during incubation under both Cd and Pb exposure (Table 3). Initial pH values remained near neutrality (~7.0) during the early incubation period but declined substantially after prolonged metal exposure. After 24 h, the pH decreased to 5.10±0.34 in Cd-treated cultures and 4.91±0.08 in Pb-treated cultures. The greater acidification observed under Pb exposure was consistent with the higher oxalic acid levels detected by HPLC.

**Table 3.** pH variation during exposure of *E. coli* K-12 MG1655 to 1,000 p.p.m. Cd and Pb

Time (hr)	1,000 p.p.m. Cd	1,000 p.p.m. Pb
**0**	7.0250±0.16^a^	6.9750±0.09^a^
**1**	7.0650±0.21^a^	7.0100±0.13^a^
**2**	7.1900±0.01^a^	6.9150±0.06^a^
**3**	6.9800±0.32^a^	6.8800±0.04^a^
**4**	6.8600±0.28^ab^	6.5450±0.16^b^
**5**	6.3400±0.76^ab^	5.6950±0.15^c^
**6**	6.0400±0.52^b^	5.4850±0.26^c^
**24**	5.0950±0.34^c^	4.9100±0.08^d^

Different superscript letters within each treatment indicate significant differences (LSD, *P*<0.05). pH variation of *E. coli* K-12 MG1655 exposed to 1,000 ppm Cd and Pb over 24 h. Both treatments showed a progressive decline in pH, with Pb causing greater acidification than Cd.

### FTIR evidence of oxalic acid synthesis

**Fig. 2.**
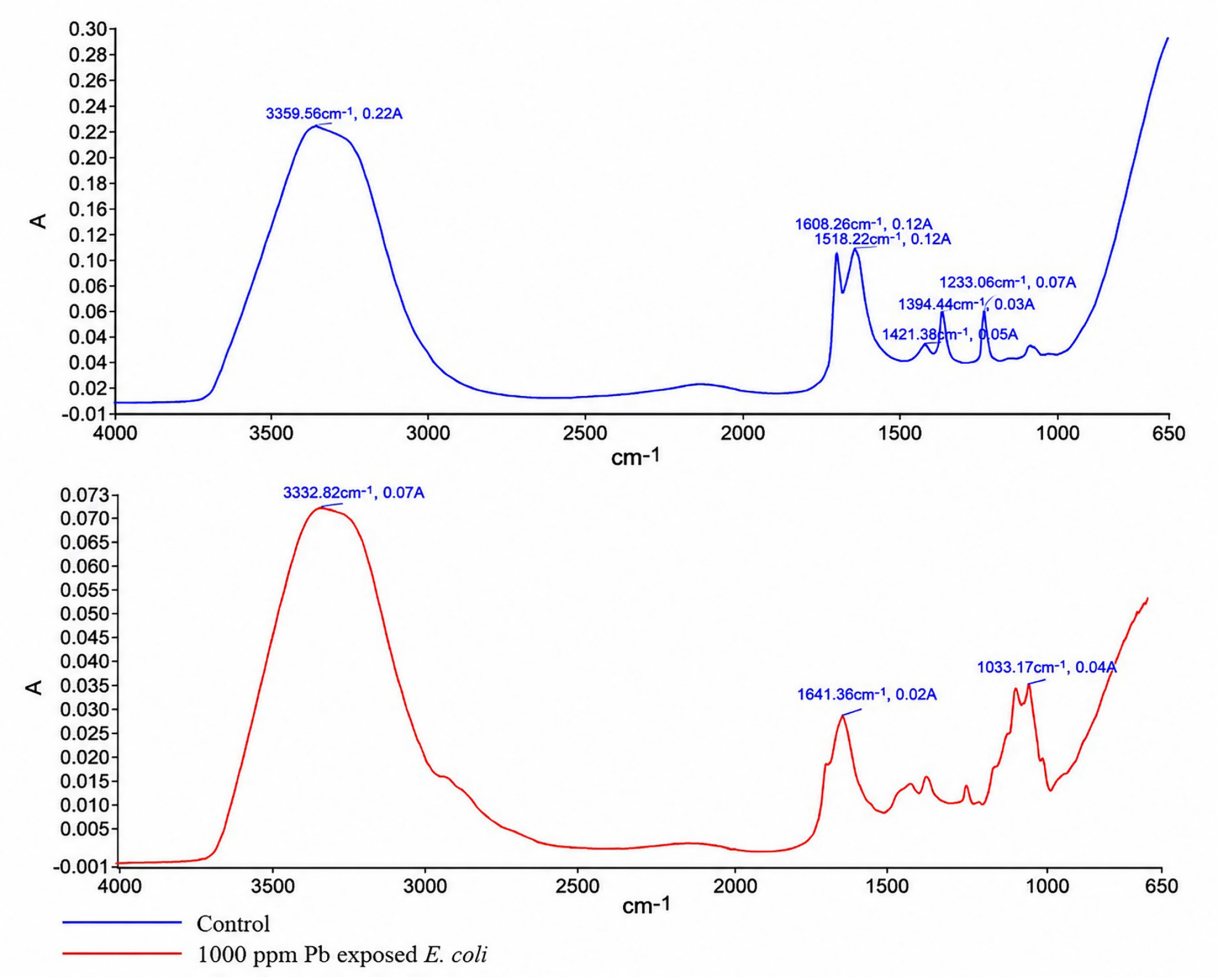
FTIR spectra of culture supernatant from control and Pb-exposed *E. coli*, showing oxalic acid-metal complexation signatures. **Fig. 2** shows FTIR spectra comparing control and Pb-exposed (1,000 p.p.m.) *E. coli* culture supernatants, showing shifts and intensity changes in O-H, carboxylate and fingerprint-region bands consistent with oxalic acid-lead complexation.

FTIR analysis of the culture supernatant showed clear spectral differences between Pb-treated samples and the control (Fig. 2). The control supernatant exhibited strong O–H/N–H stretching (3,359 cm^−1^), carboxylate vibrations (1,421 cm^−1^) and multiple bands between 1,200 and 1,000 cm^−1^ attributed to extracellular metabolites. In contrast, Pb-exposed supernatant showed markedly reduced intensities across these organic functional groups, consistent with oxalic-acid consumption during metal complexation. A distinct band at 1,033 cm^−1^ appeared exclusively in the Pb-treated supernatant, corresponding to C–O–Pb vibrations characteristic of oxalate–metal species. These findings indicate that extracellular oxalic acid synthesized by *E. coli* reacted with Pb^2+^ to form Pb-oxalate complexes detectable in the supernatant.

### Bioaccumulation efficiency of *E. coli* for Pb and Cd removal

The bioaccumulation efficiency of *E. coli* K-12 MG1655 to eliminate Pb and Cd in aqueous solutions was determined across varying metal concentrations, ranging from 100 to 1,000 p.p.m. (Table 4). The results demonstrate highly efficient biomass-associated sequestration of Pb, Cd and their combination. *E. coli* at 100 p.p.m. had a Pb removal efficiency of 99.48% and Cd removal efficiency of 81.44%. Increasing concentration to 200 p.p.m. significantly increased Cd removal to 93.76% (*P*=0.02). At 500 p.p.m., the efficiencies increased to 99.80% for Pb and 96.96% for Cd. *E. coli* were at its optimum at the highest concentration of 1,000 p.p.m., with a 99.94% removal of Pb and 97.77% of Cd. The overall removal efficiency was 98.19% for 1,000 p.p.m. Pb+Cd. These results highlight the great potential of *E. coli* in bioaccumulating Pb and Cd, with the removal of Pb always higher than the removal of Cd at all concentrations.

**Table 4.** Bioaccumulation efficiency of *E. coli* for Pb and Cd

Treatment	Mean±sd (%)
100 p.p.m. Pb	99.48±0.13^a^
100 p.p.m. Cd	81.44±3.18^b^
200 p.p.m. Pb	99.53±0.04^a^
200 p.p.m. Cd	93.76±4.18^c^
500 p.p.m. Pb	99.80±0.02^a^
500 p.p.m. Cd	96.96±1.40^cd^
1,000 p.p.m. Pb	99.94±0.02^a^
1,000 p.p.m. Cd	97.77±0.28^cd^
1,000 p.p.m. Pb+Cd	98.19±0.20^d^

Different superscript letters indicate significant differences between treatments (LSD, *P*<0.05). Bioaccumulation efficiency (%) of *E. coli* for Pb, Cd, and combined Pb + Cd at 100–1,000 ppm. Pb showed consistently high bioaccumulation efficiency (~99–100%), whereas Cd exhibited lower and more variable efficiencies.

### Desorption efficiency of Pb and Cd from *E. coli* biomass using HCl and HNO_3_

The efficiency of desorption with *E. coli* K-12 MG1655 biomass was assessed at varying concentrations (0.01 M and 0.1 M) of HCl and HNO_3_. In the case of Pb, the maximum desorption efficiency (98.5%) was achieved using 0.1M HNO_3_ and then 95.46% for 0.1M HCl. Weaker solutions (0.01M HCl and 0.01M HNO_3_) gave lower recoveries of 56.78% and 71.11%, respectively (Table 5). Cd followed the same pattern: desorption efficiency was 91.5% and 85.81% with 0.1M HNO_3_ and 0.1M HCl, respectively. Lower concentrations (0.01M HCl and 0.01M HNO_3_) had much lower recoveries of 51.78% and 64.61% (Table 6). These findings suggest that 0.1 M HNO_3_ is the most effective desorbing agent of both Pb and Cd, and the concentration and type of acid used are critical in improving the desorption of heavy metals from *E. coli* biomass.

**Table 5.** Desorption efficiency of Pb from *E. coli* biomass using different concentrations of HCl and HNO₃

Acid treatment	Desorption (%) mean±sd
0.01 HCl	56.78±1.63ᵈ
0.01 HNO₃	71.11±1.63^c^
0.01 HCl	95.46±0.81ᵇ
0.01 HNO₃	98.50±0.65ᵃ

Different superscript letters indicate significant differences between treatments (LSD, *P*<0.05). Desorption efficiency (%) of Pb from *E. coli* biomass using HCl and HNO₃ at different concentrations. Pb recovery increased with acid concentration and was higher with HNO₃ than with HCl.

**Table 6.** Desorption efficiency of Cd from *E. coli* biomass using different concentrations of HCl and HNO₃

Acid treatment	% Recovered (mean±sd)
0.01 M HCl	51.78±1.33^a^
0.01 M HNO₃	64.61±2.51^b^
0.1 M HCl	85.81±1.61^c^
0.1 M HNO₃	91.50±1.40^d^

Different superscript letters indicate significant differences between treatments (LSD, *P*<0.05). Desorption efficiency (%) of Cd from *E. coli* biomass using HCl and HNO₃ (0.01 and 0.1 M). Cd recovery increased with acid concentration and was higher with HNO₃ than with HCl.

### Desorption of Pb and Cd from *E. coli* cells using ohmic heating

The desorption efficiency of Pb and Cd from *E. coli* K-12 MG1655 cells using ohmic heating was assessed by applying a controlled electrical current to cell suspensions (Table 7). The recovery rates were measured after 10 min of treatment: Cd had a desorption efficiency of 45.83%, and Pb had a desorption efficiency of 45.38%. These findings suggest that desorption of the heavy metals in the cells of the *E. coli* bacteria can be obtained using ohmic heating, which makes this method a possible alternative to the conventional acid-based desorption procedures.

**Table 7.** Desorption efficiency of Pb and Cd from *E. coli* biomass using ohmic heating

Metal	% Recovered (mean±sd)
Cd	45.83±2.61^a^
Pb	45.38±4.38^a^

Same superscript letter indicate no significant differences between treatments (LSD, *P*<0.05). Desorption efficiency (%) of Cd and Pb from *E. coli* biomass using ohmic heating. Cd and Pb showed similar recovery efficiencies (~45%).

## Discussion

The present study demonstrates that *E. coli* K-12 MG1655 adaptation to heavy metal stress is a multifaceted biochemical and physiological process, and the production of oxalic acid is a significant detoxification pathway. The high tolerance limits observed for both Cd and Pb (Tables1  2) further support the robustness of the adaptive responses activated during metal exposure. Although tolerance assays demonstrated detectable survival of *E. coli* K-12 MG1655 at Pb and Cd concentrations above 1,000 p.p.m., oxalic–acid characterization was intentionally focused on the 1,000 p.p.m. condition because this concentration represented a severe yet biologically sustainable metal stress under which stable viability was maintained over 24 h. Oxalic acid acts as a chemical defence molecule capable of forming stable and insoluble complexes with divalent metal ions such as Pb^2+^ and Cd^2+^. Chromatographic results indicate that the production of oxalic acid increased in the Pb-exposed setup, whereas Cd exposure was associated with a moderate increase, suggesting that the production of organic acids is metal-specific and stress-responsive [28,29]. The observed reduction in culture pH during metal exposure further supports this interpretation, as secretion of low-molecular-weight organic acids during microbial stress responses is commonly associated with progressive extracellular acidification. Organic acids have been reported in detoxification by microbial taxa; oxalate is produced by species including *Aspergillus niger* and *Bacillus subtilis* to precipitate toxic metals in inert minerals [30,31]. This comparative evidence indicates that oxalic acid secreted by the culture of *E. coli* stabilizes metal ions through the formation of the oxalate complex and, therefore, decreases oxidative stress and preserves intracellular structures. In real-world systems, micro-organisms that generate organic acids are often enriched in soils contaminated with metals, where the precipitation of metal oxalate is a critical component of the natural detoxification process [32,33]. As a result, the increased production of oxalic acid at Pb exposure does not only substantiate a biochemical stress response but also places *E. coli* in the context of microbial strategies of adapting to toxic environments in general, and the argument that metabolite-based detoxification is central to microbial metal tolerance.

FTIR analysis of the Pb-treated supernatant revealed a marked attenuation of major organic functional group bands relative to the control, together with the emergence of a distinct absorption at ~1,033 cm^−1^. This spectral pattern is consistent with extracellular oxalic acid reacting with Pb^2+^ to form Pb–oxalate species, which consume free carboxylate groups and generate characteristic C–O–Pb vibrational modes in the 1,030–1,040 cm^−1^ region [34,35]. Vibrational reference data further indicate that oxalate and metal–oxalate salts exhibit strong absorptions near 1,310–1,320 cm^−1^ and ~1,030 cm^−1^, supporting assignment of the new peak to oxalate–Pb coordination [36,37]. Biologically, the formation of such complexes aligns with established microbial detoxification strategies, as many bacteria and fungi secrete oxalic acid under metal stress and immobilize metals through precipitation of insoluble oxalate phases [32,35]. Collectively, these spectral changes provide evidence that extracellular oxalic acid produced by *E. coli* actively mediates Pb complexation in the supernatant, contributing to Pb immobilization via formation of Pb–oxalate species.

Although HPLC and FTIR analyses consistently supported the presence of oxalate-associated responses during metal exposure, the present metabolite analysis was intentionally focused on high-concentration stress conditions and relied on a single external oxalic-acid calibration standard. Consequently, the current data should be interpreted as supportive evidence of oxalate-associated detoxification rather than exhaustive quantitative characterization of oxalate metabolism. Future work involving multi-point calibration, concentration-dependent profiling and temporal metabolomic analysis would further clarify the relationship between oxalate secretion, metal speciation, and uptake dynamics.

Building on this biochemical observation, a crucial point concerns the mechanistic link between oxalic acid secretion and subsequent metal uptake. The high bioaccumulation efficiencies (99.94% of Pb and 97.77% of Cd at 1,000 p.p.m.) indicate that *E. coli* has an outstanding ability to bind and retain metal ions. This is in line with known facts that metal biosorption occurs through a primary interface with the bacterial cell envelope, which contains electronegative functional groups in abundance [38,39]. When considered together with the oxalic acid profiles, these findings imply that oxalate does not merely detoxify but may enhance metal binding by altering local chemical environments. Organic acids can acidify microenvironments, increase ligand availability and form bridging complexes that stabilize metal species at the cell surface. The potential role of microbial low-molecular-weight organic acids in metal complexation has been reported [40]. Thus, while oxalic acid formation primarily reduces toxicity, it can also lead to metal uptake by enhancing the probability of complexation or nucleation processes at the cell envelope. This aligns with the studies that report that detoxification and uptake interact in an integrated manner with extracellular metabolites determining the effectiveness of bioaccumulation [41,42].

The high Pb and Cd removal efficiencies likely reflect multiple sequestration mechanisms rather than solely intracellular accumulation. Since ICP-OES was performed on recovered biomass after centrifugation and acid digestion, the measured metals may include intracellularly accumulated, surface-bound, EPS-associated and extracellular metal–oxalate precipitated fractions co-sedimenting with the biomass. FTIR evidence of Pb–oxalate complexation further suggests that extracellular precipitation contributes under high-metal conditions. Thus, ‘bioaccumulation’ here refers broadly to biomass-associated sequestration involving both biosorptive and intracellular processes. Future studies using sequential extraction, electron microscopy with elemental mapping or compartment-specific analyses are needed to distinguish these mechanisms.

Bioaccumulation in *E. coli* likely involves multiple complementary mechanisms operating at both the cell surface and intracellular levels. The Gram-negative cell envelope contains abundant negatively charged functional groups, including carboxyl, phosphate and hydroxyl moieties within lipopolysaccharides, membrane proteins and peptidoglycan, which facilitate rapid electrostatic binding of divalent metal ions, such as Pb^2+^ and Cd^2+^ [43]. In addition, *E. coli* can produce EPS under environmental stress, creating a hydrated extracellular matrix capable of trapping and immobilizing metals through adsorption and complexation reactions [44]. EPS-associated polysaccharides and proteins provide additional ligand-binding sites that may enhance sequestration efficiency while simultaneously limiting direct intracellular toxicity. Following initial surface interactions, a proportion of metal ions may subsequently enter the cell through nonspecific transport systems or damaged membrane regions, where intracellular sequestration and detoxification processes further contribute to metal retention [45]. The high removal efficiencies observed in the present study, therefore, likely reflect a combined contribution of surface adsorption, EPS-associated immobilization, extracellular oxalate-mediated complexation/precipitation and intracellular accumulation rather than a single uptake mechanism alone.

Additional evidence of this argument includes structural and biochemical findings of previous studies, including FTIR-detected protein, polysaccharide and carboxyl group vibration variations [46] and SEM-observed changes in cell-surface under metal stress [47]. The latter changes are consistent with previous studies that showed that metabolic stress induces EPS reorganization and exposure to functional groups, hence, altering metal affinity [48]. These data provide conceptual evidence that detoxification metabolites and cell-surface chemistry operate in tandem to optimize uptake. The present results, therefore, contribute not only experimental confirmation of high uptake rates but also mechanistic depth explaining how metabolite-augmented biosorption could occur in *E. coli* under Pb and Cd stress.

This study further highlights the high bioaccumulation efficiency displayed by *E. coli* relative to other microbial systems. Removal rates of Pb and Cd of up to 99% and 98%, respectively, put *E. coli* at a very high-performance level of known biosorptive micro-organisms. As an example, fungi like *Aspergillus* usually exhibit Pb removal efficiencies of 80–90% at similar concentrations [49], whereas many bacterial systems, including *Pseudomonas aeruginosa* or *B. subtilis*, usually exhibit Cd removal of 60–85% under similar conditions [50,51]. Some of the highly touted biosorbents, such as *Saccharomyces cerevisiae*, rarely reach a 90% removal of Pb in single-metal systems [52]. The relatively good performance of *E. coli* in the current study can be explained by its physiological plasticity, rapid growth and strong responses to stress, which have long been used as a model organism in metabolic studies [53,54]. Importantly, in mixed-metal systems, where competitive inhibition often suppresses biosorption capacity, *E. coli* still retained >98% removal efficiency, indicating strong resilience to competing ions. Given that environmental contamination rarely involves single-metal exposure, this robustness under multi-metal stress enhances the ecological validity of *E. coli* as a bioremediation agent. Linking back to the aim of this study, these results strengthen the hypothesis that *E. coli* is metabolically capable as well as operationally beneficial in comparison to more specialized microbial systems.

The differential removal behaviour observed between Pb and Cd likely reflects differences in metal physicochemical properties and their interaction with bacterial binding sites. Pb^2+^ generally exhibits a stronger affinity for negatively charged functional groups such as carboxyl, phosphate and hydroxyl moieties present on bacterial cell envelopes and EPS, which may contribute to its consistently higher sequestration efficiency relative to Cd ^2+^ [55,56]. In addition, Pb forms highly stable oxalate complexes with relatively low solubility, potentially enhancing extracellular immobilization under the conditions tested. Despite the coexistence of both metals in the mixed-metal system, overall removal efficiency remained exceptionally high, suggesting that competitive inhibition was limited and that the available binding and complexation capacity of the system was not saturated. Similar observations have been reported in bacterial biosorption systems where living microbial biomass retained substantial sequestration capacity under multimetal exposure due to the combined effects of surface adsorption, EPS-mediated binding and metabolite-assisted complexation [57,58]. The persistence of high removal efficiency under binary-metal exposure further suggests that oxalate-associated detoxification and biomass-surface interactions may operate synergistically to buffer competitive ion effects [58]. This behaviour is environmentally significant because contaminated industrial effluents, mining drainage and electronic-waste leachates rarely contain single-metal pollutants in isolation. Therefore, the sequestration performance observed under Pb+Cd co-exposure supports the applicability of the *E. coli*-based system to chemically complex real-world remediation scenarios.

These findings indicate that oxalate synthesis in *E. coli* K-12 MG1655 is not an incidental stress by-product but part of a coordinated adaptive programme. Our findings, therefore, do not simply demonstrate oxalate production as an isolated metabolic artefact but place it within the broader regulatory framework of stress adaptation in *E. coli*, involving coordinated oxidative stress signalling and carbon-flux re-routing. This strengthens the argument that oxalate-mediated detoxification represents a regulatable and potentially engineerable trait, increasing its value for biotechnology, where predictable responses in controlled production systems are essential.

Another critical component of this study centres on biorecovery, an aspect mostly overlooked in conventional biosorption research. The results of desorption efficiency indicate that there is a distinct order of performance in the release of the metals in terms of recovery: 0.1 M HNO_3_ was the most effective in releasing the metals (98.5% of Pb and 91.5% of Cd) followed by 0.1 M HCl, whereas lower concentrations of the two acids were significantly (*P*=0.013) less effective. These results correspond to the chemical behaviour of metal-biomass interactions with stronger acids having higher proton-exchange capacity and destabilizing metal-ligand complexes more efficiently [59,60]. The high performance of nitric acid may be explained by its oxidative nature, which helps to break down biomolecular matrices and release metal ions. Nevertheless, acid desorption is efficient; however, it increases sustainability concerns. Acid use generates secondary waste streams and introduces corrosion risks, limiting industrial applicability unless recovery circuits are tightly controlled. Therefore, the inclusion of ohmic heating is particularly significant. This physicochemical technique provides a chemical-free, energy-driven alternative, which is in line with the concept of green technology, although its recovery efficiencies were moderate compared to acid desorption. Ohmic heating has also been suggested as a possible technique to disinfect wastewater, and the method is already used in food processing, where it has the benefit of being easy to manage and scale [27,61, 62]. In a circular bioeconomy model, which is defined by recovery, reuse and the minimum amount of waste generation, this strategy has a significant potential following further optimization. As a result, biorecovery findings not only provide quantitative data but also conceptual information on how microbial bioremediation can be integrated into sustainable industrial processes.

Acid desorption and ohmic heating present different operational and sustainability trade-offs. Dilute HNO₃ was particularly effective for releasing biomass-associated Pb and Cd, likely due to enhanced proton exchange and partial disruption of metal–ligand interactions within the biomass matrix [63]. However, acid-based regeneration approaches require continuous chemical input and may generate secondary acidic waste streams requiring downstream neutralization and disposal, in addition to introducing potential corrosion and material-compatibility challenges during scale-up [64]. By contrast, ohmic heating provided moderate recovery efficiencies under the present conditions but offers advantages as a chemical-minimized recovery strategy driven primarily by electrical energy input. Because heating occurs volumetrically through electrical resistance, ohmic systems may provide relatively rapid and controllable biomass disruption with lower chemical dependency and reduced secondary waste generation [65]. Although the recovery efficiency observed here remained lower than acid desorption, the operational conditions used were intentionally preliminary and non-optimized. Further refinement of voltage gradients, reactor conductivity, treatment duration and electrode configuration could potentially improve metal release efficiency and biomass regeneration performance. From a circular bioeconomy perspective, the possibility of partially regenerating and reusing microbial biomass while minimizing chemical consumption may represent an important advantage for future large-scale remediation systems treating complex metal-containing effluents.

The final point of analysis concerns the environmental significance and translational potential of the integrated detoxification–uptake–recovery model demonstrated in this study. Heavy metal contamination continues to be a critical ecological and population-health problem globally [66]. Traditional methods of treatment, which include precipitation, ion exchange and electrochemical remediation, are expensive and have low efficiency at dilute concentrations [6,67]. The current results suggest that microbial systems would be a viable alternative: not only does the system of microbes detoxify and accumulate metals with a high level of efficiency but also allows the recovery of the metals. This is a twofold ability that is necessary in the development of circular remediation systems that would be able to attain both environmental purification and resource recovery. A microbial system capable of simultaneous sequestration and recovery may potentially reduce operational costs and chemical input in future remediation applications, although economic feasibility and process scalability remain to be validated at pilot and industrial scales. In addition, the metabolic interdependence between oxalic-acid synthesis and metal uptake is observed to give a mechanistic roadmap to strain engineering in the future. By optimizing the microbial recovery pathways further, membrane engineering or transporter overexpression may be a possibility through increased organic-acid secretion. Real-world analogues exist in wastewater bioreactors and bioleaching operations, where microbial consortia are engineered to improve metal capture and yield. Therefore, the current work provides evidence of the feasibility of the concept under consideration, as well as outlines some of the future avenues of practical implementation.

**Fig. 3.**
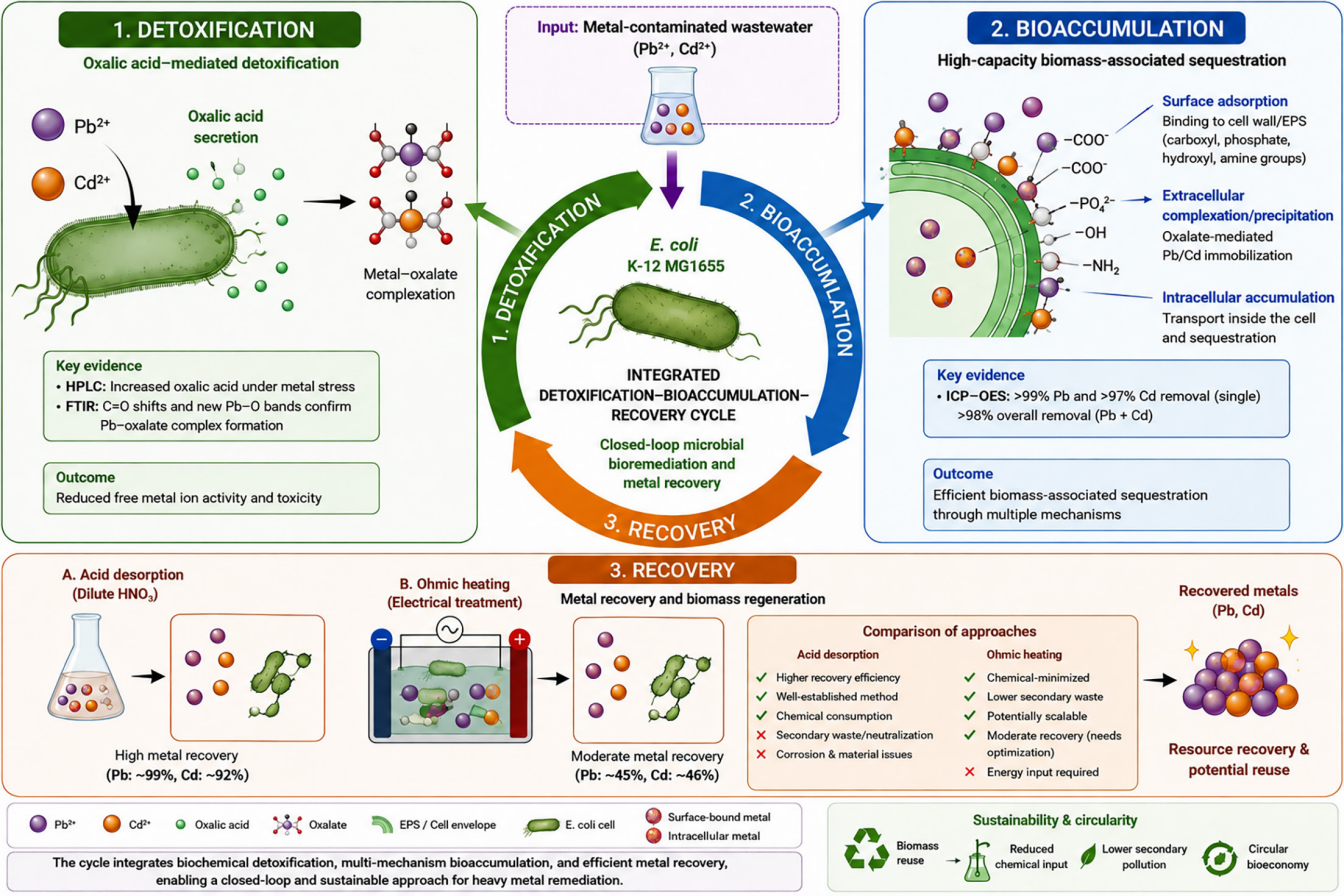
Integrated detoxification–bioaccumulation–recovery cycle of Pb and Cd processing by *E. coli* K-12 MG1655. The schematic summarizes Pb and Cd processing by *E. coli* under metal stress. Oxalic acid secretion promotes extracellular metal complexation and detoxification, while biomass-associated sequestration occurs via surface adsorption, extracellular precipitation/complexation and intracellular accumulation. Biomass-associated metals are subsequently recovered through acid desorption or ohmic heating-assisted regeneration, highlighting the potential of integrated circular bioremediation systems linking detoxification, sequestration and metal recovery.

Taken together, our results move beyond conventional biosorption reports that treat detoxification, removal and recovery as disconnected operations. By integrating oxalate-mediated detoxification, high-capacity bioremoval and subsequent metal recovery within a single *E. coli* K-12 MG1655 platform (Fig. 3), we demonstrate a coherent workflow for managing Pb and Cd at high loadings. The inclusion of ohmic heating as a complementary regeneration strategy broadens the technological relevance of the system by demonstrating the feasibility of chemical-minimized recovery approaches alongside conventional acid desorption. This integrated perspective underscores the potential of *E. coli*-based processes as flexible, regenerable tools for heavy metal management.

## Conclusion

This study provides an integrated evaluation of detoxification, metal sequestration and recovery in *E. coli* K-12 MG1655 under Pb and Cd stress. The organism produced detectable levels of oxalic acid under Pb and Cd exposure, with Pb eliciting the strongest response, underscoring the role of oxalate in mitigating toxicity through complexation and precipitation of metal ions. This biochemical adaptation was highly consistent with the extremely high bioaccumulation potential of the organism, where both metals and nearly full uptake of Pb were removed at the highest levels. Such performance highlights the strong capacity of *E. coli* K-12 MG1655 for biomass-associated Pb and Cd sequestration under high-metal conditions. Given this performance under controlled laboratory conditions, the system may provide a promising basis for future evaluation in scaled bioreactor configurations, including packed-bed or fluidized-bed systems, following further optimization and pilot-scale validation.

In parallel, the study established viable recovery pathways capable of reclaiming the sequestered metals from the biomass. The optimum recovery was obtained using 0.1M HNO_3_, where the maximum percentage of Pb and Cd was 98.5% and 91.5%, respectively, which means that biosorbents can be chemically regenerated. Though the recovery rates of ohmic heating were relatively low, its chemical-free and scalable characteristics make it preferable to use as an environmentally friendly solution, which can be optimized to be used in industry.

In summary, these findings demonstrate an integrated microbial remediation–recovery framework in which detoxification, metal sequestration and subsequent recovery are functionally linked within a single biological system. Collectively, the results support the potential suitability of *E. coli* K-12 MG1655 as a candidate organism for future development of closed-loop bioremediation strategies targeting Pb- and Cd-contaminated environments.

## Abbreviations

EPS: extracellular polymeric substance
FTIR: Fourier-transform infrared characterization
ICP-OES: inductively coupled plasma–optical emission spectrometry
LSD: least significant difference
MSM: mineral salt medium

## References

[1] World Health Organization (2007). Health risks of heavy metals from long-range transboundary air pollution.

[2] European Commission (2000). Directive 2000/60/EC of the European Parliament and of the Council establishing a framework for Community action in the field of water policy.

[3] United States, Environmental Protection Agency, Office of Resources Management (1979). Legislation, programs and organization--the United States Environmental Protection Agency.

[4] Centers for Disease Control and Prevention (2001). Agency for Toxic Substances and Disease Registry. Strategic plan for public health workforce development. Toward a life-long learning system for public health practitioners.

[5] Mukpo A (2023). As Shell, Eni quit Niger Delta, state-backed report describes legacy of carnage.

[6] Gunatilake S (2015). Methods of removing heavy metals from industrial wastewater. Methods.

[7] Nnaji ND, Onyeaka H, Miri T, Ugwa C (2023). Bioaccumulation for heavy metal removal: a review. SN Appl Sci.

[8] Carolin CF, Kumar PS, Saravanan A, Joshiba GJ, Naushad M (2017). Efficient techniques for the removal of toxic heavy metals from aquatic environment: a review. J Environ Chem Eng.

[9] Wang Z, Zhang Y, Chen Y, Han F, Shi Y (2024). Competition of Cd(II) and Pb(II) on the bacterial cells: a new insight from bioaccumulation based on NanoSIMS imaging. Appl Environ Microbiol.

[10] Sarkodie EK, Jiang L, Li K, Yang J, Guo Z (2022). A review on the bioleaching of toxic metal(loid)s from contaminated soil: insight into the mechanism of action and the role of influencing factors. Front Microbiol.

[11] Joshi S, Gangola S, Bhandari G, Bhandari NS, Nainwal D (2023). Rhizospheric bacteria: the key to sustainable heavy metal detoxification strategies. Front Microbiol.

[12] Huang Y, Zhang L, Yuan S, Liu W, Zhang C (2023). The production of oxalate by *Aspergillus niger* under different lead concentrations. Agronomy.

[13] Asunmo MY, Ogunnusi TA, Akpor OB (2024). 2024 International Conference on Science, Engineering and Business for Driving Sustainable Development Goals (SEB4SDG).

[14] George F, Mahieux S, Daniel C, Titécat M, Beauval N (2021). Assessment of Pb (II), Cd (II), and Al (III) removal capacity of bacteria from food and gut ecological niches: insights into biodiversity to limit intestinal biodisponibility of toxic metals. Microorganisms.

[15] Ali Q, Ayaz M, Yu C, Wang Y, Gu Q (2022). Cadmium tolerant microbial strains possess different mechanisms for cadmium biosorption and immobilization in rice seedlings. Chemosphere.

[16] Rizvi A, Ahmed B, Zaidi A, Khan MS (2020). Biosorption of heavy metals by dry biomass of metal tolerant bacterial biosorbents: an efficient metal clean-up strategy. Environ Monit Assess.

[17] Rathnayake Mudiyanselage HDR (2025). Application of chelating agents in micronutrient recovery.

[18] Chiang SM, Schellhorn HE (2012). Regulators of oxidative stress response genes in *Escherichia coli* and their functional conservation in bacteria. Arch Biochem Biophys.

[19] Joudeh N, Saragliadis A, Schulz C, Voigt A, Almaas E (2021). Transcriptomic response analysis of *Escherichia coli* to palladium stress. Front Microbiol.

[20] Rui B, Shen T, Zhou H, Liu J, Chen J (2010). A systematic investigation of *Escherichia coli* central carbon metabolism in response to superoxide stress. BMC Syst Biol.

[21] Jiang Y, Ge F, Li F, Zhang D, Deng S (2020). Intracellular metabolomics switching alters extracellular acid production and insoluble phosphate solubilization behavior in *Penicillium oxalicum*. Metabolites.

[22] Chowdhury NB, Schroeder WL, Monteiro L, Burnum-Johnson KE (2025). mGem: revisiting bacterial overflow metabolism. mBio.

[23] Grąz M (2024). Role of oxalic acid in fungal and bacterial metabolism and its biotechnological potential. World J Microbiol Biotechnol.

[24] Bhatia RP, Kirit HA, Predeus AV, Bollback JP (2022). Transcriptomic profiling of *Escherichia coli* K-12 in response to a compendium of stressors. Sci Rep.

[25] LaVoie SP, Summers AO (2018). Transcriptional responses of *Escherichia coli* during recovery from inorganic or organic mercury exposure. BMC Genomics.

[26] Maynaud G, Brunel B, Mornico D, Durot M, Severac D (2013). Genome-wide transcriptional responses of two metal-tolerant symbiotic *Mesorhizobium* isolates to zinc and cadmium exposure. BMC Genomics.

[27] Chu H-YI, Zhang X, Wang Y, Miri T, Onyeaka H (2025). Sustainable extraction of bioactive compounds from cocoa shells waste and brewer's spent grain using a novel two-stage system integrating ohmic-accelerated steam distillation (OASD) and supercritical CO2 extraction (SSCO2). Sustainability.

[28] Gomathy M, Sabarinathan K (2010). Microbial mechanisms of heavy metal tolerance-a review. Agri Rev.

[29] Jain D, Kour R, Bhojiya AA, Meena RH, Singh A (2020). Zinc tolerant plant growth promoting bacteria alleviates phytotoxic effects of zinc on maize through zinc immobilization. Sci Rep.

[30] Ghosh S, Rusyn I, Dmytruk OV, Dmytruk KV, Onyeaka H (2023). Filamentous fungi for sustainable remediation of pharmaceutical compounds, heavy metal and oil hydrocarbons. Front Bioeng Biotechnol.

[31] Sazanova (nee Barinova) KV, Frank-Kamenetskaya OV, Vlasov DYu, Zelenskaya MS, Vlasov AD (2020). Carbonate and oxalate crystallization by interaction of calcite marble with *Bacillus subtilis* and *Bacillus subtilis*–*Aspergillus niger* association. Crystals.

[32] Fomina M, Hillier S, Charnock JM, Melville K, Alexander IJ (2005). Role of oxalic acid overexcretion in transformations of toxic metal minerals by *Beauveria caledonica*. Appl Environ Microbiol.

[33] Newsome L, Falagán C (2021). The microbiology of metal mine waste: bioremediation applications and implications for planetary health. Geohealth.

[34] National Institute of Standards and Technology (2025). NIST Chemistry WebBook, SRD 69: lead oxalate (C₂O₄Pb).

[35] Zhang L, Song X, Shao X, Wu Y, Zhang X (2019). Lead immobilization assisted by fungal decomposition of organophosphate under various pH values. Sci Rep.

[36] Frost RL, Yang J, Ding Z (2003). Raman and FTIR spectroscopy of natural oxalates: Implications for the evidence of life on Mars. ChinSciBull.

[37] Ou X, Zhang F, Wang C (2012). Degradation of orange G induced by Fe(III)-oxalate complex in irradiated solution. Asian J Chem.

[38] González Henao S, Ghneim-Herrera T (2021). Heavy metals in soils and the remediation potential of bacteria associated with the plant microbiome. Front Environ Sci.

[39] Naja GM, Volesky B (2017). Handbook of Advanced Industrial and Hazardous Wastes Management.

[40] Zheng X, Lin H, Du D, Li G, Alam O (2024). Remediation of heavy metals polluted soil environment: a critical review on biological approaches. Ecotoxicol Environ Saf.

[41] Diep P, Mahadevan R, Yakunin AF (2018). Heavy metal removal by bioaccumulation using genetically engineered microorganisms. Front Bioeng Biotechnol.

[42] Presentato A, Piacenza E, Turner RJ, Zannoni D, Cappelletti M (2020). Processing of metals and metalloids by *Actinobacteria*: cell resistance mechanisms and synthesis of metal(loid)-based nanostructures. Microorganisms.

[43] Fein JB, Yu Q, Nam J, Yee N (2019). Bacterial cell envelope and extracellular sulfhydryl binding sites: their roles in metal binding and bioavailability. Chem Geol.

[44] Zhou X, Kang F, Qu X, Fu H, Alvarez PJJ (2020). Role of extracellular polymeric substances in microbial reduction of arsenate to arsenite by *Escherichia coli* and *Bacillus subtilis*. Environ Sci Technol.

[45] Gupta P, Diwan B (2017). Bacterial exopolysaccharide mediated heavy metal removal: a review on biosynthesis, mechanism and remediation strategies. *Biotechnol Rep*.

[46] Wang Z, Tan R, Gong J, Gong B, Guan Q (2023). Process parameters and biological mechanism of efficient removal of Cd(II) ion from wastewater by a novel *Bacillus subtilis* TR1. Chemosphere.

[47] Renu S, Sarim KM, Singh DP, Sahu U, Bhoyar MS (2021). Deciphering cadmium (Cd) tolerance in newly isolated bacterial strain, *Ochrobactrum intermedium* BB12, and its role in alleviation of Cd stress in spinach plant (*Spinacia oleracea* L.). Front Microbiol.

[48] Zhang H, Zhang J, Tang S, Deng Z, Makar RS (2025). Exopolysaccharide-producing strains alter heavy metal fates and bacterial communities in soil aggregates to reduce metal uptake by pakchoi. Front Microbiol.

[49] de Wet MMM, Brink HG (2021). Lead biosorption characterisation of *Aspergillus piperis*. Sustainability.

[50] Sinha S, Mukherjee SK (2009). *Pseudomonas aeruginosa* KUCD1, a possible candidate for cadmium bioremediation. Braz J Microbiol.

[51] Xie Y, He N, Wei M, Wen T, Wang X (2021). Cadmium biosorption and mechanism investigation using a novel *Bacillus subtilis* KC6 isolated from pyrite mine. J Clean Prod.

[52] Zinicovscaia I, Yushin N, Abdusamadzoda D, Grozdov D, Shvetsova M (2020). Efficient removal of metals from synthetic and real galvanic zinc-containing effluents by brewer's yeast *Saccharomyces cerevisiae*. Materials.

[53] Qin W, Zhao J, Yu X, Liu X, Chu X (2019). Improving cadmium resistance in *Escherichia coli* through continuous genome evolution. Front Microbiol.

[54] Worden CR, Kovac WK, Dorn LA, Sandrin TR (2009). Environmental pH affects transcriptional responses to cadmium toxicity in *Escherichia coli* K-12 (MG1655). FEMS Microbiol Lett.

[55] Qu C, Yang S, Mortimer M, Zhang M, Chen J (2022). Functional group diversity for the adsorption of lead(Pb) to bacterial cells and extracellular polymeric substances. *Environ Pollut*.

[56] Lu S, Li X, Xi Y, Liu H, Zhang Z (2021). Insight the roles of loosely-bound and tightly-bound extracellular polymeric substances on Cu2+, Zn2+ and Pb2+ biosorption process with *Desulfovibrio vulgaris*. J Colloid Interface Sci.

[57] Lin H, Wang C, Zhao H, Chen G, Chen X (2020). A subcellular level study of copper speciation reveals the synergistic mechanism of microbial cells and EPS involved in copper binding in bacterial biofilms. Environ Pollut.

[58] Shi X, Ling Q, Jiang Z, Pei F, Xin M (2023). Insight into the roles of soluble, loosely bound and tightly bound extracellular polymeric substances produced by *Enterobacter* sp. in the Cd^2+^ and Pb^2+^ biosorption process: characterization and mechanism. Colloids Surf B Biointerfaces.

[59] Usmonkulova A, Malusa E, Kadirova G, Khalilov I, Canfora L (2025). Ni^2+^ and Cd^2+^ biosorption capacity and redox-mediated toxicity reduction in bacterial strains from highly contaminated soils of Uzbekistan. Microorganisms.

[60] Yanaka M, Nagoya S, Kawase Y (2025). A novel dynamic biosorption kinetic model based on reactions on the biosorbent surface for biosorption of heavy metal copper by non-living biomass waste tea leaves. Environ Surfaces Interfaces.

[61] Alkanan ZT, Altemimi AB, Al-Hilphy ARS, Watson DG, Pratap-Singh A (2021). Ohmic heating in the food industry: developments in concepts and applications during 2013–2020. *Appl Sci*.

[62] Javed T, Oluwole-ojo O, Zhang H, Akmal M, Breikin T (2025). System design, modelling, energy analysis, and industrial applications of ohmic heating technology. Food Bioprocess Technol.

[63] Hammaini A, González F, Ballester A, Blázquez ML, Muñoz JA (2007). Biosorption of heavy metals by activated sludge and their desorption characteristics. J Environ Manage.

[64] Ali Redha A (2020). Removal of heavy metals from aqueous media by biosorption. *Arab J Basic Appl Sci*.

[65] Indiarto R, Rezaharsamto B (2020). A review on ohmic heating and its use in food. Int J Sci Technol Res.

[66] Ali MM, Hossain D, Khan MS, Begum M, Osman MH (2021). Heavy Metals-Their Environmental Impacts and Mitigation.

[67] Qasem NAA, Mohammed RH, Lawal DU (2021). Removal of heavy metal ions from wastewater: a comprehensive and critical review. *NPJ Clean Water*.

